# Hypertension and physical activity in middle-aged and older adults in China

**DOI:** 10.1038/s41598-018-34617-y

**Published:** 2018-10-31

**Authors:** Yinghui You, Wenjie Teng, Jincai Wang, Guifeng Ma, Anning Ma, Junjie Wang, Pengtao Liu

**Affiliations:** 1 Weifang Medical University, Weifang, Shandong Province China; 2 Chinese Center for Disease Control and Prevention (China CDC), Beijing, China

## Abstract

There are few studies examining the association between levels of physical activity and hypertension in middle-aged and older adults in China. Data were drawn from the Chinese Health and Retirement Longitudinal Study (using four-stage stratified probability-proportional-to-size sampling), involving 7113 individuals aged 45 years and older from 28 provinces of China. Hypertension was defined as a systolic BP ≥ 130 mm Hg, or diastolic BP ≥ 80 mm Hg, or self-reported use of anti-hypertensive medications. The awareness, treatment, and control among hypertensive participants were 53.12%, 43.37%, and 10.03%, respectively. The prevalence of hypertension was 56.12% among all the participants, higher in main city zones (58.68%) than villages (55.52%) and other areas (55.78%, p < 0.0001). Participants who were overweight (BMI ≥ 24: AOR 4.08, 95% CI 3.21–5.20, P < 0.0001; BMI ≥ 28: 10.03, 7.56–13.31, P < 0.0001), and drinking more than once a month (1.28, 1.12–1.46, P < 0.0001) were more likely to have hypertension. The decision tree model was established to analyze the importance of different levels of physical activity on hypertension prevention. Participants who usually participated in moderate-to-vigorous activity for more than 10 minutes (vigorous: 0.82, 0.73–0.91, P = 0.0004; moderate: 0.83, 0.75–0.92, P = 0.0006) were less likely to have hypertension. The results of the decision tree showed that the vigorous physical activity seemed to be more important than moderate and light activity to induce beneficial effects on prevention of hypertension. The strength of our study is in using the decision tree to clearly rank the importance of those key factors affecting hypertension.

## Introduction

Hypertension is one of the leading risk factors for cardiovascular diseases, which has aroused more and more people’s attention all over the world. In this study, a novel hypertension BP threshold definition compared with the Chinese Health and Retirement Longitudinal Study (CHARLS) data from 2015 was adopted in order to bring people bigger awareness of prevention of hypertension. Translation into clinical practice poses a huge challenge for clinicians in China due to its large population by the end of 2017. The new guidelines for hypertension were released by the American College of Cardiology/American Heart Association (ACC/AHA) at threshold BP ≥ 120/80 mm Hg and targeting a BP < 130/80 mm Hg^1^.

According to the China Patient-Centered Evaluative Assessment of Cardiac Events (PEACE) 2017, the prevalence of hypertension (BP ≥ 140/90) was 44.7% among Chinese adults aged 35–75 years, of whom 30.1% were taking prescribed antihypertensive medications, and only 7.2% had achieved control^2^. In the past decades, changes in lifestyle have accompanied China’s rapid social and economic development. Some adverse changes (such as decreased physical activity, less sleep, irregular diet) may have increased the risk of hypertension^3,4^. The new hypertension guidelines suggest that patients with hypertension should be treated at least with non-pharmacological measures^1^. While blood pressure can usually be best regulated through lifestyle modifications before it reaches the stage of hypertension, there is a range of treatment options^5^. Lifestyle adjustments, including physical activity and exercise, are the standard first-line treatments for hypertension^6^. It is well known that favorable physiological changes and overall health benefits are associated with regular physical activity^7^. Together with evidence from observational and clinical studies, appropriate levels of physical activity may reduce the occurrence of hypertension through complex biological pathways^6–9^.

Although several investigators have evaluated influences of physical activity on hypertension prevention, few have assessed the optimal duration (for how long), frequency (how often), volume (how much in total) and intensity (how hard) of doing physical activity, that would lead to lower or normal blood pressure among middle-aged and older adults. To fill this gap in knowledge, we completed a cross-sectional study to evaluate the relation between levels of physical activity and hypertension.

## Results

### The prevalence of hypertension among the study participants

Among 7 113 participants in this study, the prevalence of hypertension (systolic blood pressure, SBP ≥ 130 OR diastolic blood pressure, DBP ≥ 80) was 56.12%, higher in main city zones (58.68%) than villages (55.52%) and other areas (55.78%; p < 0.0001). The awareness, treatment, and control among hypertensive participants were 53.12%, 43.37%, and 10.03%, respectively. The prevalence was hgher among drinkers than non-drinkers and increased with age and body mass index (BMI) (Table 1).

**Table 1:** The weighted hypertension prevalence of the study participants (n = 7113).

Variables	%Hypertension*	P
Total	56.12	
Awareness (%)	53.12	
Taking medicine (%)	43.37	
Control (%)	10.03	
Age		<0.0001
45–54	44.02	
55–64	56.20	
65–74	66.18	
≥75	73.71	
Gender		0.0882
Male	57.25	
Female	55.07	
BMI (kg/m^2^)		<0.0001
<18.5	39.12	
18.5–23.9	47.54	
24–27.9	62.38	
≥28	77.85	
Smoking status		0.4787
Never	57.05	
Ever or current	55.49	
Drinking frequency		0.0094
Never	51.30	
≤1/month	58.79	
>1/month	55.74	
Sleep duration		0.3212
<7 h sleep	56.98	
7–10 h sleep	55.23	
≥10 h sleep	56.94	
Residency (%)		<0.0001
Main city zone	58.68	
Others	55.78	
Village	55.52	
<4 Hours	51.37	
≥4 Hours	47.54	

### Results of logistic regression models describing the association between lifestyle behaviors and hypertension

After adjusting for demographics, behavioral risks, and current residence, participants who were overweight (BMI ≥ 24) (AOR 4.08, 95% CI 3.21–5.20, P < 0.0001), obese (BMI ≥ 28) (10.03, 7.56–13.31, P < 0.0001), and drank more than once a month (1.28, 1.12–1.46, P < 0.0001) were more likely to have hypertension (Table 2). On the other hand, participants who usually took part in vigorous or moderate activity more than 10 minutes every week (vigorous: 0.82, 0.73–0.91, P = 0.0004; moderate: 0.83, 0.75–0.92, P = 0.0006) were less likely to have hypertension (Table 3).

**Table 2:** Associations between lifestyle behaviors and hypertension.

Variables	OR (95% CI)	P	AOR (95% CI)	P
Age				
45–54	1.00		1.00	
55–64	1.63 (1.46,1.83)	<0.0001	1.87 (1.66,2.11)	<0.0001
65–74	2.49 (2.18,2.84)	<0.0001	2.98 (2.58,3.44)	<0.0001
≥75	3.57 (2.97,4.29)	<0.0001	5.55 (4.46,6.91)	<0.0001
Gender				
Male	1.00		1.00	
Female	0.92 (0.83,1.01)	0.0649	0.99 (0.85,1.15)	0.8497
BMI (kg/m^2^)				
<18.5	1.00		1.00	
18.5–23.9	1.41 (1.14,1.74)	<0.0001	1.92 (1.52,2.43)	<0.0001
24–27.9	2.58 (2.08,3.20)	<0.0001	4.08 (3.21,5.20)	<0.0001
≥28	5.47 (4.24,7.05)	<0.0001	10.03 (7.56,13.31)	<0.0001
Smoking status				
Never	1.00		1.00	
Ever or current	1.07 (0.97,1.17)	0.1921	1.05 (0.91,1.22)	0.5060
Drinking frequency				
Never	1.00		1.00	
≤1/month	0.84 (0.71,0.98)	0.0031	0.88 (0.73,1.05)	0.0040
>1/month	1.13 (1.02,1.26)	0.0006	1.28 (1.12,1.46)	<0.0001
Sleep duration				
<7 h sleep	1.00		1.00	
7–10 h sleep	0.93 (0.84,1.03)	0.2020	0.98 (0.88,1.09)	0.9209
≥ 10 h sleep	1.00 (0.85,1.18)	0.6730	0.95 (0.79,1.14)	0.6311
Residence				
Village	1.00		1.00	
Main city zone	1.14 (1.00,1.29)	0.0623	1.01 (0.88,1.16)	0.9675
Other	1.01 (0.89,1.15)	0.4305	1.03 (0.89,1.20)	0.7327

**Table 3:** Multivariate analysis of the hypertension risk factors associated with activity behaviours and patterns.

Variables	%Hypertension	OR (95% CI)	P	AOR (95% CI)	P
Taking part in activity more than 10 minutes a week					
Inactive		1.00		1.00	
Vigorous	49.61	0.70 (0.63,0.77)	<0.0001	0.82 (0.73,0.91)	0.0004
Moderate	52.42	0.73 (0.66,0.80)	<0.0001	0.83 (0.75,0.92)	0.0006
Light	55.74	0.97 (0.87,1.10)	0.6537	0.99 (0.87,1.13)	0.9000
Time usually spend doing activity ≥30 minutes one day					
Inactive		1.00		1.00	
Vigorous	55.28	1.33 (0.88,2.02)	0.1782	1.11 (0.71,1.74)	0.6470
Moderate	52.59	0.97 (0.78,1.19)	0.7473	0.93 (0.74,1.16)	0.5152
Light	56.58	0.91 (0.78,1.07)	0.2606	0.90 (0.75,1.07)	0.2273
Time usually spend doing activity ≥2 hours one day					
Inactive		1.00		1.00	
Vigorous	48.70	0.91 (0.75,1.11)	0.3501	1.03 (0.83,1.28)	0.7934
Moderate	51.72	0.92 (0.81,1.04)	0.1944	1.00 (0.87,1.14)	0.9580
Light	53.69	0.86 (0.77,0.95)	0.0050	0.93 (0.83,1.05)	0.2222
Time usually spend doing vigorous activity ≥4 Hours one day					
Inactive		1.00		1.00	
Vigorous	47.54	0.83 (0.69,1.01)	0.0589	0.92 (0.75,1.13)	0.4160
Moderate	48.91	0.81 (0.68,0.96)	0.0179	0.87 (0.72,1.05)	0.1561
Light	51.27	0.84 (0.70,0.99)	0.0422	0.93 (0.77,1.12)	0.4135

### Importance of physical activity levels on prevention of hypertension

**Figure 1:**
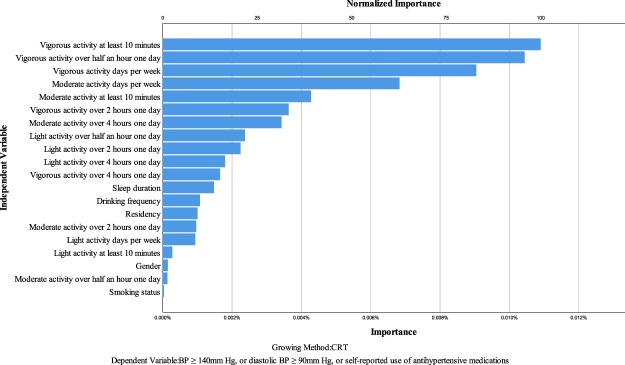
Independent variable importance on prevention of hypertension.

Figure 1 shows the independent variable importance on prevention of hypertension through the decision tree analyses. In order to increase the accuracy, sensitivity, and specificity of the model, the cutoff value of dependent variable was set to: systolic BP ≥ 140 mm Hg, or diastolic BP ≥ 90 mm Hg, or self-reported use of anti-hypertensive medications. After adjusting for BMI, which was already recognized as one of the key factors affecting the hypertension, the most important independent variable on prevention of hypertension was vigorous activity at least 10 minutes per week, and its value of normalized importance was set to 100%. The second important independent variable was vigorous activity over half an hour one day (normalized importance: 95.8%), followed by vigorous activity days per week (normalized importance: 83.0%), moderate activity over half an hour one day (normalized importance: 62.7%), moderate activity at least 10 minutes per week (normalized importance: 39.2%). The other important independent variables included vigorous activity over 2 hours one day (normalized importance: 33.3%), moderate activity over 4 hours one day (normalized importance: 31.4%), light activity over half an hour one day (normalized importance: 21.7%), light activity over 2 hours one day (normalized importance: 20.6%), light activity over 4 hours one day (normalized importance: 16.5%), sleep duration (normalized importance: 13.5%), etc. The detailed rank of the importance of physical activity levels on prevention of hypertension can be found in Fig. 1.

**Figure 2:**
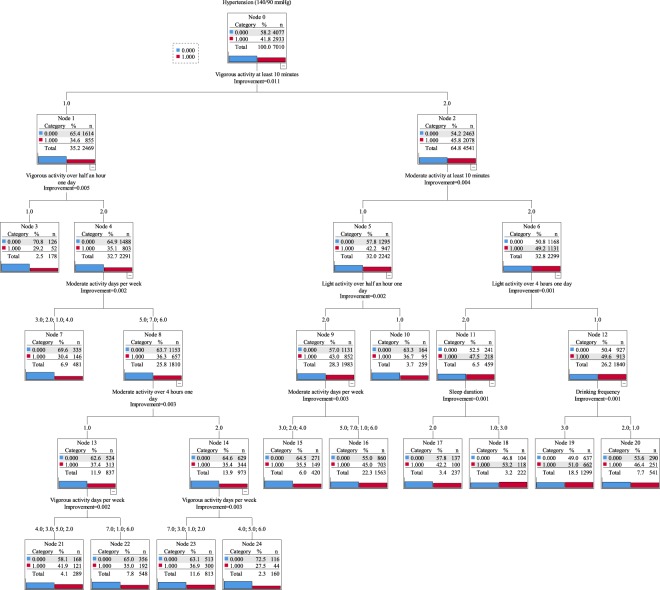
The structure of the decision tree model. Red categories indicate the prevalence of hypertension and blue categories indicate the prevalence of no hypertension. Vigorous/moderate/light activity at least 10 minutes, vigorous/moderate/light activity over half an hour/2 hours/4 hours one day (1: Yes, 2: No); Sleep duration (1: <7 hours, 2: 7–10 hours, 3: ≥10 hours); Drinking frequency (1: Never, 2: <1/month 3: >1/month); Residence (1: Main city zone, 2: Other, 3: Village); Gender (1: Male, 2: Female); Smoking status (1: Ever or current, 2: Never). Hypertension was defined as systolic BP ≥ 140 mm Hg, or diastolic BP ≥ 90 mm Hg, or self-reported use of antihypertensive medications (old definition) in this diagram.

Figure 2 shows the structure of the decision tree model. A total of 7010 participants were included in the model and 103 participants were excluded due to missing important independent variables (such as, vigorous activity at least 10 minutes, etc.). The diagram indicated that participants who usually took part in vigorous physical activity 4–6 days every week (less than half an hour one day), and moderate physical activity 5–7 days every week (less than 4 hours one day), had the lowest prevalence of hypertension (27.5%, Node 24). On the other hand, participants who had shorter (<7 hours) or longer (≥10 hours) sleep duration, never took part in light physical activity more than 4 hours one day per week, and never took part in vigorous or moderate physical activity the whole week had the highest prevalence of hypertension (53.2%, Node 18).

## Discussion

In this nationally representative longitudinal survey of middle-aged and older adults in China, the adjusted prevalence of hypertension was 56.12%. The awareness, treatment, and control among hypertensive participants were 53.12%, 43.37%, and 10.03%, respectively. In view of new selected hypertension BP threshold definition (stage 1 hypertension starting at systolic or diastolic BP values of ≥130 mm Hg or ≥80 mm Hg), the hypertension prevalence estimate is much higher than previous studies^10^. The new hypertension guidelines with lower threshold and blood pressure targets are consistent with the World Hypertension League (WHL) global health goals with a focus on population-based risk reduction with the lowering of blood pressure^1,11^. Assuming the new hypertension guidelines are adopted in China, the high prevalence and poor control will arouse more urgent attention on hypertension. Such speed of China’s epidemiological transition will call for Chinese health department to spare no expense to decrease the high prevalence of hypertension. To better prevent and control the diseases caused by hypertension, there is a critical need for the implementation of the ACC/AHA guideline 2017 in China which is intended to increase the detection and treatment of hypertension in China.

Logistic regression indicated that participants who were overweight (BMI ≥ 24: AOR 4.08, 95% CI 3.21–5.20, P < 0.0001; BMI ≥ 28: 10.03, 7.56–13.31, P < 0.0001), and drinking more than once a month (1.28, 1.12–1.46, P < 0.0001) were more likely to have hypertension. Participants who usually took part in vigorous or moderate activity more than 10 minutes every week (vigorous: 0.82, 0.73–0.91, P = 0.0004; moderate: 0.83, 0.75–0.92, P = 0.0006) were less likely to have hypertension. So we suggested patients pay more attention to proper physical activity to achieve a normal weight to decrease the rate of hypertension.

The strength of our study is in using a decision tree which clearly ranks the importance of those key factors affecting hypertension. As is shown in the tree model, vigorous activity at least 10 minutes per week, vigorous activity over half an hour one day, and days of vigorous activity per week were the three most important factors for preventing hypertension. Clearly, vigorous physical activity was far more effective than moderate or light activity on prevention of hypertension. In addition, days of vigorous or moderate physical activity per week was also important.

The results of the decision tree model suggest that even 10 minutes of vigorous physical activity every week can help reduce the prevalence of hypertension. This was consistent with previous studies that increasing physical activity to recommended levels (aerobic activity should be performed in bouts of at least 10 minutes duration) would help eliminate 6% to 10% of the major non-communicable diseases^12,13^. For additional health benefits, we recommend that older adults increase their vigorous physical activity to half an hour one day. This recommendation would differ in cases where an older adult cannot take part in any vigorous physical activity. In addition, there are other various types of physical activities for them to choose from. As the node 10 of the decision tree showed, the prevalence of hypertension was 36.7%, which was below the average; in this node the participants just took part in moderate activity more than half an hour and also light activity more than half an hour one day (Fig. 2). Although the decision tree model could not reflect the specific patterns, it could be a helpful reference for undertaking a new fitness regime for hypertension prevention. It was worth noting that vigorous physical activity would increase the risk of musculoskeletal injury rates. In order to avoid such a risk, it’s better to encourage adults aged 45 years and older to start physical activity from moderate to higher levels gradually.

The prevalence of hypertension was 58.68% among participants living in main city zones, higher than participants living in villages (55.52%). There are several possible factors that could result in the difference. Firstly, it has been well-documented that most rural residents in China had to spend more time on hard manual labor because most of them were self-employed in agriculture-related activities^14^. More time doing hard manual labor would reduce the prevalence of hypertension in villagers. Secondly, risk factors of hypertension such as growing rates of stress, irregular diet, irregular physical activity, smoking, obesity, and pollution were more serious in main city zones than in villages^15^. For that reason, public health campaigns and incentives need to be strengthened in main city zones of China.

In this study, 47.91% of the participants was male and 52.09% were female. The prevalence of hypertension did not present significant gender differences overall: 57.25% for males and 55.07% for females (P = 0.0882). A previous study showed that the prevalence of hypertension was higher among males until the age of 45, nearly equal between the two genders from 45 to 64 years old, and after that hypertension became more prevalent among women^16^. In our study, the interaction of age and gender may be the reason why there was no significant difference in the prevalence of hypertension between two genders. Further studies will be needed to confirm the effect of gender on hypertension among Chinese older adults.

Our study had several potential limitations. First, the cross-sectional nature of our study provided limited evidence for the causal association between physical activity and hypertension. We simply offer suggestions for physical activity to prevent hypertension according to the observed results. We did not conduct a long-term experiment to identify the number of weeks of exercise required to observe a significant reduction in hypertension. Second, the machine learning of decision tree generally shows very good results in various “theoretical” environments. However, in real life it often lacks the ability to measure attribute values that the traditional method does. Human behavior is much more complicated than the model simulation of the real world, and this conclusion is worthy of clinical application and further research. Third, genetic factors are another important factor associated with hypertension in China. Many related studies had been performed in China and the results showed that human cytochrome P450 had the most important influence on the development of essential hypertension in China^17–19^. However, no consideration was given to this factor in this study because of the lack of genetic information. Fourth, the novel US guidelines from 2017 to analyze the CHARLS data from 2015 (the definition for hypertension at that time in China was systolic BP ≥ 140 mm Hg, or diastolic BP ≥ 90 mm Hg) would inevitably produce selection bias. The proportion of anti-hypertensive treatment and effectiveness according to the 2017 US guidelines might have little significance considering that the anti-hypertensive treatment in 2015 was based on the 2015 valid national guidelines. However, the present study relates to the 2017 guidelines which are not sufficient in China. This study may provide a reference for future research. New research will be required to solve these issues.

However, these limitations do not considerably affect our study findings and recommendations for the prevention of hypertension. Our study highlights the importance of vigorous physical activity on prevention of hypertension among adults aged 45 years and older in China, which can raise people’s awareness of insisting on such physical activity.

## Methods

### Ethical approvals

Ethical approval for the study was granted by the Ethical Review Committee of Peking University. All methods of this study were performed in accordance with the relevant guidelines and regulations. Written informed consent was obtained from all study participants.

### Study design and study participants

This cross-sectional study was based on the China Health and Retirement Longitudinal Study (CHARLS) survey conducted by the National School of Development at Peking University. CHARLS was patterned after the Health and Retirement Study (HRS) in the USA, the Survey of Health, Ageing, and Retirement in Europe (SHARE) and the English Longitudinal Study of Ageing (ELSA). Multi-stage stratified probability-proportional-to-size sampling method was used to draw the sample^20^. Demographic characteristics, socioeconomic status, lifestyle behaviors, and health status were collected by face-to-face interviews^21^. The detailed design and methods of the CHARLS study had been described previously^22,23^. Data were drawn from the fourth wave of CHARLS which was conducted in 2015 (released in October 2017, version ID: 20171011), consisting of individuals aged 45 years and older from 28 provinces of China who completed assessments of healthy lifestyle behaviors and measurement of hypertension. The questionnaires were recovered on the spot at the same time. BP was measured by sphygmomanometer 3 times at 5-minute intervals. Physical exercise practice was calculated according to self-reported information through household interviews.

### Definitions

BP of the participants was measured by a trained physician with the electronic sphygmomanometer (Omron HEM-7200 Monitor, Batteries, and Stopwatch). The participants in this study were required to rest in a seated position for at least 5 minutes before the BP measurement, and the BP was measured 3 times at 5-minute intervals. The mean of the 3 readings was calculated. The threshold of hypertension in the current valid guidelines for hypertension in China was systolic BP ≥ 140 mm Hg, or diastolic BP ≥ 90 mm Hg. In order to raise people’s urgent attention to the risk of hypertension, compared hypertension was defined as a systolic BP ≥ 130 mm Hg, or diastolic BP ≥ 80 mm Hg, or self-reported use of antihypertensive medications in the past 2 weeks irrespective of the BP according to the ACC/AHA guideline 2017 in this study. Awareness of hypertension was defined by a self-report of a prior diagnosis of hypertension by a doctor. Treatment of hypertension was defined as the self-reported use of taking Chinese traditional medicine, taking Western modern medicine, or other treatments for the management of hypertension. Control of hypertension was defined as the pharmacological treatment of hypertension with an average SBP < 130 mmHg and an average DBP < 80 mmHg.

The participants who indicated that they drank any alcoholic beverages, such as beer, wine, or liquor in the past year were identified as ‘drinkers’, and drinkers were asked about their drinking frequency. The participants who reported drinking any type of alcoholic beverage more than once a month were classified as ‘>1/month’ drinkers and those who had drank but less than once a month were classified as ‘≤1/month’ drinkers.

The participants were asked the question “Now we would like to ask about the amount of time you spend on different types of physical activities in a usual week”. Light physical activity was defined as walking to travel from place to place, and any other walking that you might do solely for recreation, sport, exercise, or leisure. Moderate physical activity was defined as making breath somewhat harder than normal and may include carrying light loads, bicycling at a regular pace, or mopping the floor. Vigorous activity was defined as making breath much harder than normal and may include heavy lifting, digging, plowing, aerobics, fast bicycling, or cycling with a heavy load.

### Statistical analysis

Statistical analysis was performed using R 3.4.1 (https://www.r-project.org/) and took into account the sample weights and complex survey design of the CHARLS. There are two sets of cross-section weights released in this wave of CHARLS, one without non-response adjustment, and another with. We used the cross-section weights with non-response adjustment of the participants in this study, and the construction method of this weights is similar to the baseline weights but taking account of the death and divorce^22^. The *chisq.test* function in stats package of R were used to compare categorical variables between subjects with and without hypertension, respectively. The associations between categorical variables were tested using contingency tables and the χ^2^ test. Logistic regression was used to examine the lifestyle factors related to the development of hypertension, and the *glm* function in stats package of R was used to fit these models. All statistical tests were two-tailed, and a p-value < 0.05 was considered statistically significant.

### Decision tree analysis

Decision tree model was used to analyze the normalized importance of different physical activity levels on prevention of hypertension. Rpart (R package, version 4.1–13) and SPSS statistics (version 22.0) was used to create the model. We used the decision tree model not only for their primary task (the decision making), but also for outlining the normalized importance of each independent variable using a well-known property of the decision trees—their knowledge representation. Previous studies had demonstrated that being overweight or obese increased the likelihood of developing hypertension^5,24,25^. In this study, BMI was selected as an influence variable which defines how much influence an individual has on the tree-growing process. Individuals with lower influence values have less influence; and individuals with higher values have more. Our main interest was to discover the independent variable importance (different levels of physical activity) on prevention of hypertension. Classification regression tree (CART) growing method was used to split the data into segments that were as homogeneous as possible with respect to the dependent variable. The sum of goodness for each split was used to measure of the variable importance in the decision tree model^26,27^.

## Data Availability

All data in CHARLS are maintained at the National School of Development of Peking University and will be accessible to researchers around the world at the study website. http://charls.pku.edu.cn/zh-CN/page/data.
